# Fabrication of graphene films with high transparent conducting characteristics

**DOI:** 10.1186/1556-276X-8-440

**Published:** 2013-10-23

**Authors:** Xiying Ma, Hao Zhang

**Affiliations:** 1 School of Mathematics and Physics, Suzhou University of Science and Technology, 1# Kerui Road, Suzhou, Jiangsu 215009, China

**Keywords:** Graphene film, Transparent conducting characteristics, Sheet resistance, Transparency

## Abstract

We present a study on the transparent conducting characteristics of graphene-based films prepared by means of rapid chemical vapor deposition. The graphene films were grown on quartz slides with a CH_4_/Ar mixed gas under a constant flow at 950°C and then annealed at 1,000°C. It was found that the graphene films present excellent electrical conductivity with high transparency. The conductivity is up to 1,240 S/cm, the sheet resistance is lower than 1 kΩ/sq, and the transparency is well over 85% in the visible wavelength range of 400 to 800 nm, showing that the graphene films have very low resistivity and superior transparency and completely satisfy the need for transparent conductors. These properties can be used in many applications, such as transparent conductor films for touch panels.

**PACS:**

61.48.+c, 78.67.Pt, 68.37.Hk, 68.65.Ac

## Background

A transparent conducting (TC) electrode is a key component in various optoelectronic devices, such as liquid crystal displays (LCDs), solar cells, organic solar cells, organic light-emitting diodes (OLEDs), etc. [1-4]. Indium tin oxide (ITO) is widely used as a transparent conducting electrode for these devices, but it is costly and shows poor transparency in the blue and near-infrared light ranges, instability in the presence of acids or bases, and susceptibility to ion diffusion into the substrate [5,6]. Graphene exhibits an excellent carrier electronic mobility property [7,8] and high transparency for visible and near-infrared spectra. Moreover, it is abundant in source and cheap in price, nontoxic, and harmless to people and environment. It can be adopted as a transparent conducting electrode in optoelectronic devices [9,10]. For example, Wu et al. reported graphene as a TC electrode for organic LED [11]. Also, Gan et al. and Ye et al. reported CdSe nanoribbon (NR)/graphene Schottky solar cells [12,13].

In using graphene as a TC electrode, it is very important to deposit a large-scale uniform graphene film on Si and other substrates. Graphene has been deposited in various approaches, such as chemical vapor deposition (CVD) [14], metal-based epitaxy [15,16], and other technologies [17,18]. Recently, there have been reports on noncomposite reduction of graphene oxide (GO) into graphene using chemical routes and high-temperature annealing [19,20]. It allows uniform and controllable deposition of reduced graphene oxide thin films with thicknesses ranging from a single monolayer to several layers over large areas. However, it causes some drawbacks, such as five- and seven-membered ring topological defects, which will bring down the electric conductivity of graphene. CVD has been successfully used to synthesize large-scale, conductive, and transparent graphene films from catalytic reactions that can be transferred onto arbitrary substrates [9,11]. For example, large-area graphene or few-layer graphene films on metal substrates such as Ni and Cu by CVD technology [21,22] have been reported. Since the graphene film is commonly placed on SiO_2_ and other transparent insulators in fabricating optoelectronic device architectures, graphene films on Ni or Cu must be transferred to SiO_2_ and other transparent insulator substrates, which may perplex the preparation process and technique of devices. In this work, the objective of our research was to fabricate large-area graphene films on SiO_2_ substrates and investigate their conductivity and transparency. Graphene on SiO_2_ can be easily used to make optoelectronic devices and freely transferred to other substrates by etching the SiO_2_ layer using HF. It is especially interesting for the purpose of constructing electrodes. Herein, we describe a simple and reproducible method to uniformly deposit a few layers of graphene films grown by CVD. We investigated the influence of deposition time and thickness on the transparent conducting characteristics: conductivity, sheet resistance, and transparency, of graphene films. It was found that the deposited large-scale, conductive, and highly transparent graphene films are suitable for use as constructing electrodes.

## Methods

The graphene films were fabricated on quartz crystalline slides by a rapid CVD process. The growth system was composed of a large horizontal quartz tube furnace, a vacuum system, a gas meter, and an automatic temperature controller. Quartz crystalline substrates with a size of 15 × 15 × 2 mm^3^ were cleaned ultrasonically with a sequence of acetone, ethanol, and deionized water, and then they were blown with N_2_ to dry them and placed at the center of the furnace. Prior to deposition, the furnace was pumped to 10^-2^ Pa and heated to 300°C for 10 min to remove any water moisture. High-purity CH_4_ gas (99.999%) and Ar gas with a volume ratio of 1:10 were introduced into the reactive chamber at the same temperature (950°C). In the graphene deposition process, CH_4_ was initially decomposed to give a mixture of C and H_2_, and the C atoms were condensed on the quartz substrates to form graphene films while the working pressure was kept at 50 Pa. The growth process was carried out for 1 ~ 5 min, and then the samples were annealed at 1,000°C for 20 min. Finally, when the system had cooled down to room temperature, the samples were removed.

The morphology and structure of the samples were characterized by atomic force microscopy (AFM). The structure was analyzed by Raman spectroscopy, and the optical transparency was investigated by UV–vis spectroscopy (Shimadzu UV-3600, Kyoto, Japan). Finally, the conducting characteristics of the graphene films were evaluated by Hall effect measurement (HMS-3000, Ecopia, Anyang, South Korea).

## Results and discussion

**Figure 1:**
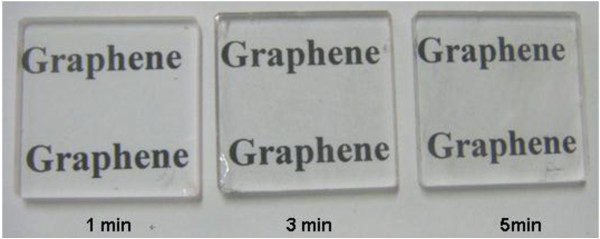
Sample pictures of graphene films on quartz substrates under different times: 1, 3, and 5 min.

**Figure 2:**
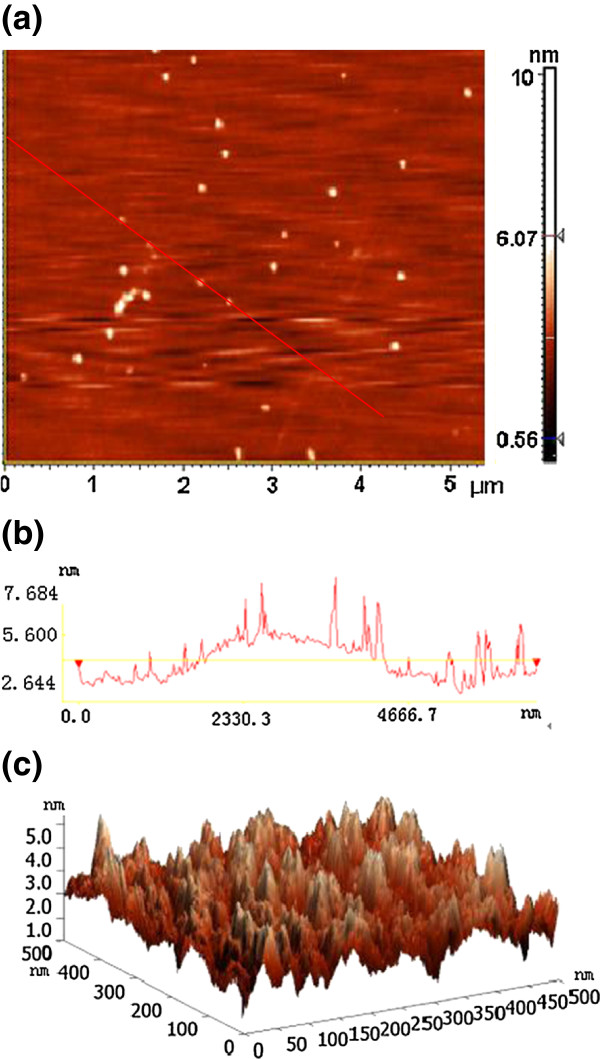
**AFM image, section analysis profile, and 3D surface morphology of the deposited graphene film. (a)** An AFM image of the graphene film deposited on quartz for 3 min. **(b)** The section analysis profile of the red line in **(a)**. The yellow horizontal line shows the position of measuring the film thickness. **(c)** 3D surface morphology of the graphene film.

Pictures of the obtained graphene films on quartz substrates under different times are shown in Figure 1. We can observe that the color of the quartz slides becomes darker with deposition time; this is because the graphene film becomes thicker with time. Figure 2a shows a typical AFM image of the graphene film deposited for 3 min. The graphene film is large scale, flat, and uniform, and only a few tiny carbon particles are scattered on it. Figure 2b shows the section analysis profile of the red line in Figure 2a. The graphene film is about 3 to 5 nm thick, and the average thickness is about 4 nm, equaling tens of layers of graphene. Figure 2c shows the three-dimensional (3D) surface morphology of the graphene film, showing its surface roughness of about 3 nm.

**Figure 3:**
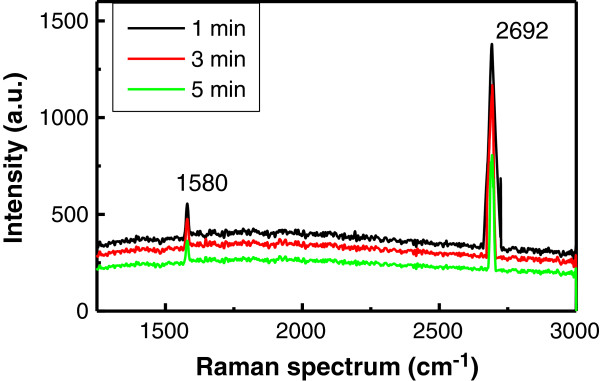
**The Raman spectra of the graphene films.** A 2D band peak at 2,692 cm^-1^ and a G band peak at 1,580 cm^-1^ are shown. The intensity ratio of the 5 min sample is *I*_D_/*I*_G_ = 2.8.

Figure 3 shows the Raman spectra of the graphene films. We can see that two major scattering peaks appear in the spectrum: a 2D band peak at 2,692 cm^-1^ and a G band peak at 1,580 cm^-1^. It is well known that the G band indicates a sound graphite carbon structure (*sp*^2^) whereas the 2D band is a typical band of graphene [23]. The disorder-induced D band (at approximately 1,350 cm^-1^) was not seen in the first-order Raman spectra. The intensity ratio of D band (*I*_D_) to G band (*I*_G_) can be used as an indication of defect quantity: a low *I*_D_*/I*_G_ corresponds to a small defect quantity. The absent D band in the Raman spectra shows that the deposited graphene in our samples has high quality. The sharp 2D peak in graphene is roughly three times (the largest intensity ratio of *I*_2D_/*I*_G_ = 2.8) more intense than the G peak, suggesting that the quality of the deposited graphene is comparable to that of graphene grown on foils [24]. The main growth mechanism of graphene on SiO_2_ with a good quality may be attributed to carbon atoms from pyrolysis of CH_4_ in the self-assembly adsorption process. Sun et al. [25] reported that carbon atoms readily arrange themselves in aromatic rings and planar *sp*^2^-hybridized graphitic layers forming nanographene on a high-temperature substrate. The second mechanism is the promotion of oxygen. Since the reactive chamber has a low ultimate vacuum pressure (about 10^-2^ Pa) in our experiment, the remaining oxygen in the tube and the high substrate temperature will promote adsorption of carbon atoms onto the quartz slide. Chen et al. [26] found that the presence of oxygen can enhance the capture of CH_*x*_ fragments through C-O and H-O binding and thus provides more opportunities for C-C coupling and graphene nucleation. Moreover, during deposition of graphene films on SiO_2_, we placed some nanoscaled Ni powder on the Si substrates in the tube to measure the electrical junction properties of graphene/Si. A few Ni nanoparticles on the Si substrates were carried on the quartz surface by CH_4_ and Ar gases, which accelerated the carbon atoms adhering and growing on the quartz, similar to that of graphene grown on Cu but not to graphene grown on Ni which occurs by a C segregation or precipitation process [21].

**Figure 4:**
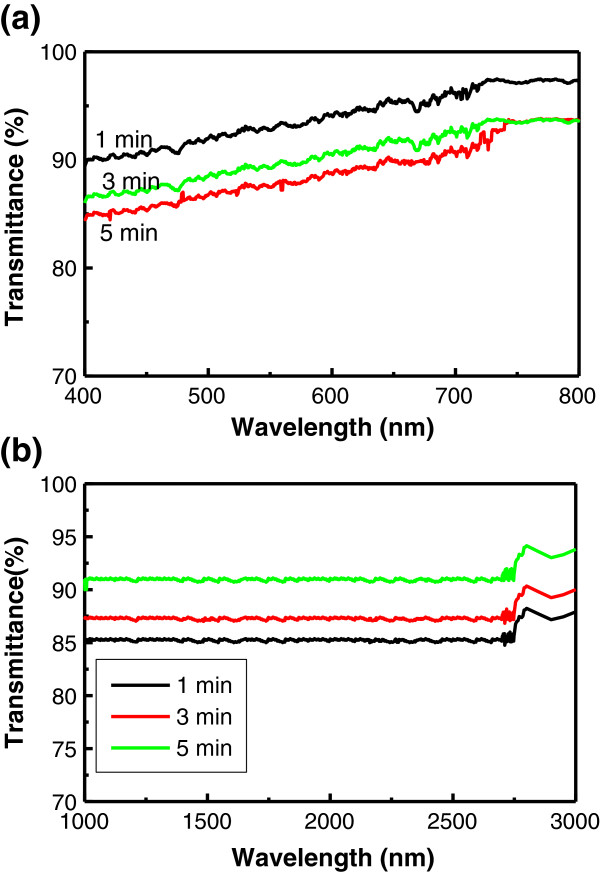
**The light transmission rate of the graphene samples. (a)** Transmission of the graphene films in the 400- to 800-nm range. **(b)** Transmission of the graphene films in the 1,000- to 3,000-nm range. The optical transmittance of the graphene films is over 85% in the visible range of 400 to 800 nm.

The visible light transmission rate of the graphene samples is shown in Figure 4a. The optical transparency value of the graphene film deposited for 1 min was very high, over 90%. However, it decreases with growth time because the film becomes thicker. On the other hand, the transparency of the 5 min sample still keeps on increasing, over 85% in the visible wavelength range of 400 to 800 nm, especially for 550 nm. Moreover, the transparency increases with wavelength. For long-wavelength light, such as in the 600- to 800-nm range, the graphene films are almost transparent. A high transmission rate is very useful for making solar cells because light in the 400- to 800-nm range has higher power. Figure 4b shows the transmission rate of the graphene samples in 1,000 to 3,000 nm near-infrared wavelength range. The transmission keeps a constant value in the near-infrared wavelength range except in the range of 2,750 to 3,000 nm, in which the transmission enhanced about 2.5%. The minimum transmission of the samples in the visible and the near-infrared range is over 85%, completely meeting the optical condition of transparent conducting films. Theoretically, the transparency of graphene drops quickly with thickness [8]. However, the actual measured transparency of graphene is not closely obeying it. For instance, Wang et al. reported that the transparency of GO is over 80% in 550-nm white light for 22 to 78 nm of thickness [27]. The high transparency of our samples is attributed to the graphene films being composed of many graphene flakes, which allowed light transmission from the tiny pits between flakes. Moreover, the pits between graphene flakes make the actual average thickness often much smaller than the measured thickness because of the resolution of the AFM instrument.

**Figure 5:**
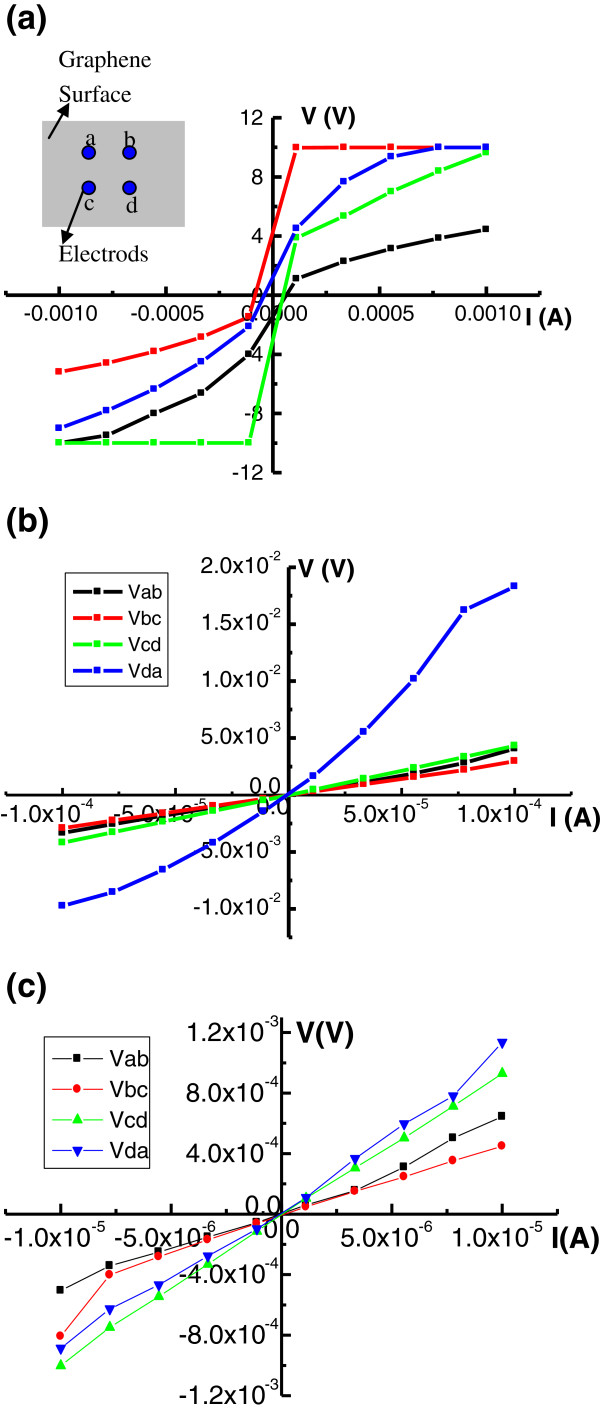
**The surface *I*-*V *behaviors of the 1, 3, and 5 min graphene samples. (a)** 1 min sample. The inset shows the electrodes’ layout on the surface of the graphene film. **(b)** 3 min sample. **(c)** 5 min sample.

The surface current–voltage (*I*-*V*) behaviors of the 1, 3, and 5 min graphene films were measured by means of Hall effect measurement, as shown in Figure 5a,b,c. The four measuring electrodes a, b, c, and d were arranged on the surface of the graphene films in a square with a side length of 1 cm, as shown the inset in Figure 5a. For the graphene deposited for 1 min, we can see that the *I*-*V* behaviors between the four points are not a characteristic of a linear relation, but of a nonlinear property. Especially, *I*-*V*_bc_ and *I*-*V*_cd_ lines were largely shifted from the linear relation. This is because the graphene on quartz does not form a continuous film but islands by a short time. With deposition time increasing to 3 and 5 min, the graphene islands collected each other to become a continuous film, and then the *I*-*V* properties become linear, as shown in Figure 5b,c. *I*-*V*_da_ in Figure 5b is far from the other lines which may be caused by the asymmetry of the four points. The *I*-*V* behaviors in Figure 5c all closely obey Ohm’s law. The linear *I*-*V* relations of the graphene surface show films with good conductivity.

**Figure 6:**
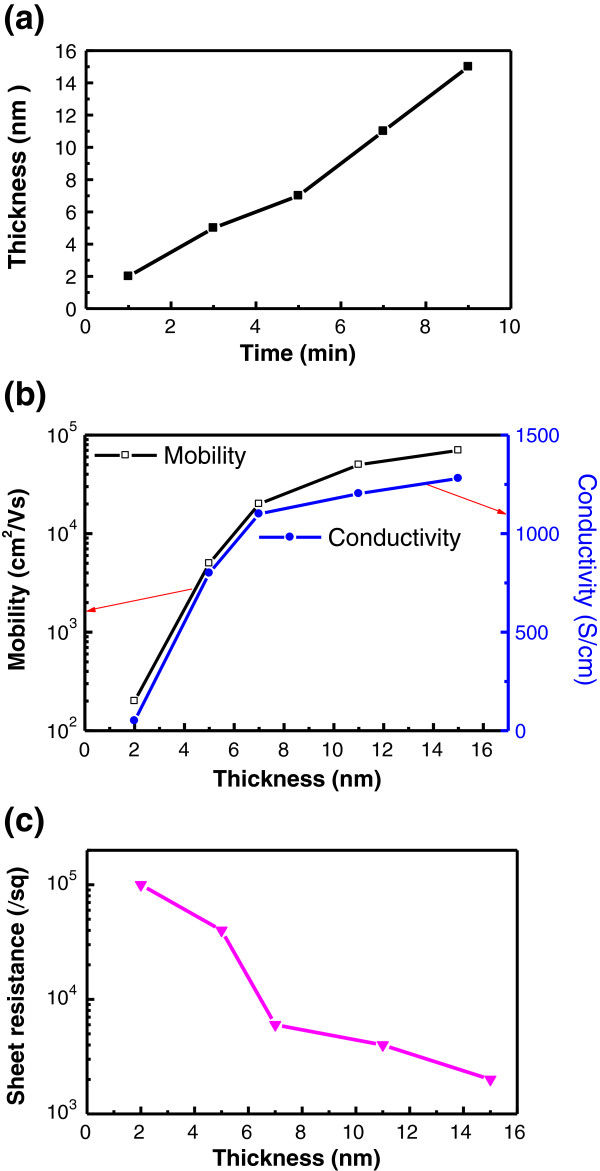
**Relation of thickness and deposition time, electron mobility, conductivity, and sheet resistance. (a)** The relation of thickness of the graphene films with deposition time. **(b)** The dependences of electron mobility and conductivity on graphene thickness. **(c)** The sheet resistance *R*_s_ changing with the thickness.

The thickness of the graphene films with deposition time is shown in Figure 6a. We can see that the thickness linearly increases with time. Then we investigated the electron mobility, conductivity, and sheet resistance with the thickness of the graphene films, as shown in Figure 6b,c. The electron mobility is 2.3 × 10^2^, 5.1 × 10^4^, and 9.5 × 10^4^ cm^2^/V/s for 1, 3, and 5 min samples, respectively. The latter two values are very close to the known ideal value of 2 × 10^5^ cm^2^/V/s [3,4]. The electron mobility and conductivity initially linearly increase and then gradually reach saturation with thickness. The results are consistent with the *I*-*V* behaviors. For a low thickness value, the graphene does not form a continuous film but many islands, which collect and fuse each other with deposition time, leading to the mobility and conductivity increasing linearly and then up to their ultimate values. The conductivity of the graphene film with a 7-nm thickness is about 1,240 S/cm, superior to that of Levendorf et al. [24] who reported 10^2^ S/cm for the same thickness. The sheet resistance *R*_s_ in Figure 6c has a reversed tendency with thickness, i.e., initially significantly drops and slowly decreases. Especially, *R*_s_ drops from 10^5^ to 10^3^ Ω/sq as the thickness increases from 2 to 7 nm. The typical *R*_s_ of the ITO film is 10^3^ ~ 10^6^ Ω/sq. Hence, the *R*_s_ of about 10^3^ Ω/sq shows that the deposited graphene has very low resistivity, satisfying the need for transparent conducting films. This value is about two times smaller than that of Wang et al. [27] who reported 2 kΩ/sq and very close to 350 Ω/sq of graphene deposited on copper then transferred on SiO_2_[22]. Wu et al. [11] reported that a graphene film with a thickness of 7 nm and a sheet resistance of 800 Ω/sq was used as a good transparent conductor of an OLED.

The graphene sample deposited for 5 min has a high transparency of over 85% in the visible wavelength range of 400 to 800 nm and a sheet resistance of 10^3^ Ω/sq. These properties are much superior to those of GO films as transparent conductors. The high performance is attributed to the CVD technique that produced compact, large-area, uniform, and high-purity graphene films.

## Conclusions

The transparent conducting properties of graphene films with different thicknesses were investigated. Ultrathin graphene films were deposited on quartz substrates by controlling a very low reactive flow rate and pressure of CH_4_ in the CVD technique. The transmission rate of the graphene films decreases with the thickness of the film, which is over 85% for the film of about 5 to 7 nm. The mobility and conductivity were found to rapidly increase up to their saturation values with the thickness of the film. The sheet resistance rapidly drops from 10^5^ to 10^3^ Ω/sq as the film thickness increases from 2 to 7 nm. The largest conductivity is up to 1,240 S/cm and the minimum sheet resistance is about 10^3^ Ω/sq, showing that the graphene films have very low resistivity and completely satisfy the need for transparent conducting films.

## Competing interests

The authors declare that they have no competing interests.

## Authors’ contributions

XM designed the structure of the graphene transistor, analyzed the results, and wrote the manuscript. HZ participated in the fabrication of the graphene films on the substrates. Both authors read and approved the final manuscript.
